# From the Cradle to the Grave: The Role of Macrophages in Erythropoiesis and Erythrophagocytosis

**DOI:** 10.3389/fimmu.2017.00073

**Published:** 2017-02-02

**Authors:** Thomas R. L. Klei, Sanne M. Meinderts, Timo K. van den Berg, Robin van Bruggen

**Affiliations:** ^1^Department of Blood Cell Research, Sanquin Research and Landsteiner Laboratory, University of Amsterdam, Amsterdam, Netherlands

**Keywords:** macrophages, red blood cells, erythropoiesis, adhesion molecules, RBC clearance, erythrophagocytosis

## Abstract

Erythropoiesis is a highly regulated process where sequential events ensure the proper differentiation of hematopoietic stem cells into, ultimately, red blood cells (RBCs). Macrophages in the bone marrow play an important role in hematopoiesis by providing signals that induce differentiation and proliferation of the earliest committed erythroid progenitors. Subsequent differentiation toward the erythroblast stage is accompanied by the formation of so-called erythroblastic islands where a central macrophage provides further cues to induce erythroblast differentiation, expansion, and hemoglobinization. Finally, erythroblasts extrude their nuclei that are phagocytosed by macrophages whereas the reticulocytes are released into the circulation. While in circulation, RBCs slowly accumulate damage that is repaired by macrophages of the spleen. Finally, after 120 days of circulation, senescent RBCs are removed from the circulation by splenic and liver macrophages. Macrophages are thus important for RBCs throughout their lifespan. Finally, in a range of diseases, the delicate interplay between macrophages and both developing and mature RBCs is disturbed. Here, we review the current knowledge on the contribution of macrophages to erythropoiesis and erythrophagocytosis in health and disease.

## Introduction

Red blood cell (RBC) turnover is a complex process that is regulated at different levels by many factors, particularly by macrophages. Macrophages shape and direct the developing RBC throughout erythropoiesis and ultimately phagocytose senescent RBCs. To perform these tasks, macrophages rely on adhesion molecules, a range of soluble and mechanical factors, and it requires cross talk with the developing or senescent RBC. Here, we review the role macrophages in erythropoiesis and erythrophagocytosis in health and disease.

## Macrophage Function in Erythropoiesis

Erythropoiesis is a highly coordinated and regulated process that consists of defined developmental stages, which are regulated by macrophages in a variety of ways. First, macrophages retain hematopoietic stem cells (HSCs) in the hematopoietic niche. Growth factors and cytokines including interleukin-3 (IL-3) and granulocyte-macrophage colony-stimulating factor (GM-CSF) facilitate proliferation and differentiation of the earliest primitive erythroid progenitor cells, so-called burst-forming unit-erythroid cells (BFU-e) (1). These slowly proliferating erythroid progenitors can then, under the influence of kidney-derived erythropoietin (Epo), differentiate into rapidly dividing colony-forming unit-erythroid cells (CFU-e) (2). Subsequent differentiation into the erythroblast stage is accompanied by the formation of so-called erythroblastic islands. Here, a central macrophage interacts with up to 30 erythroblasts through various adhesion molecules both on the macrophage as well as on the erythroblasts (3). In these erythroblastic islands, macrophages facilitate erythroblast proliferation, differentiation, and are involved in iron supply. Finally, macrophages phagocytose and digest the nuclei extruded by erythroblasts during their transition to the reticulocyte stage (4).

## Macrophages and Erythroblasts Form Erythroblastic Islands through Intercellular Adhesion Molecules

**Figure 1:**
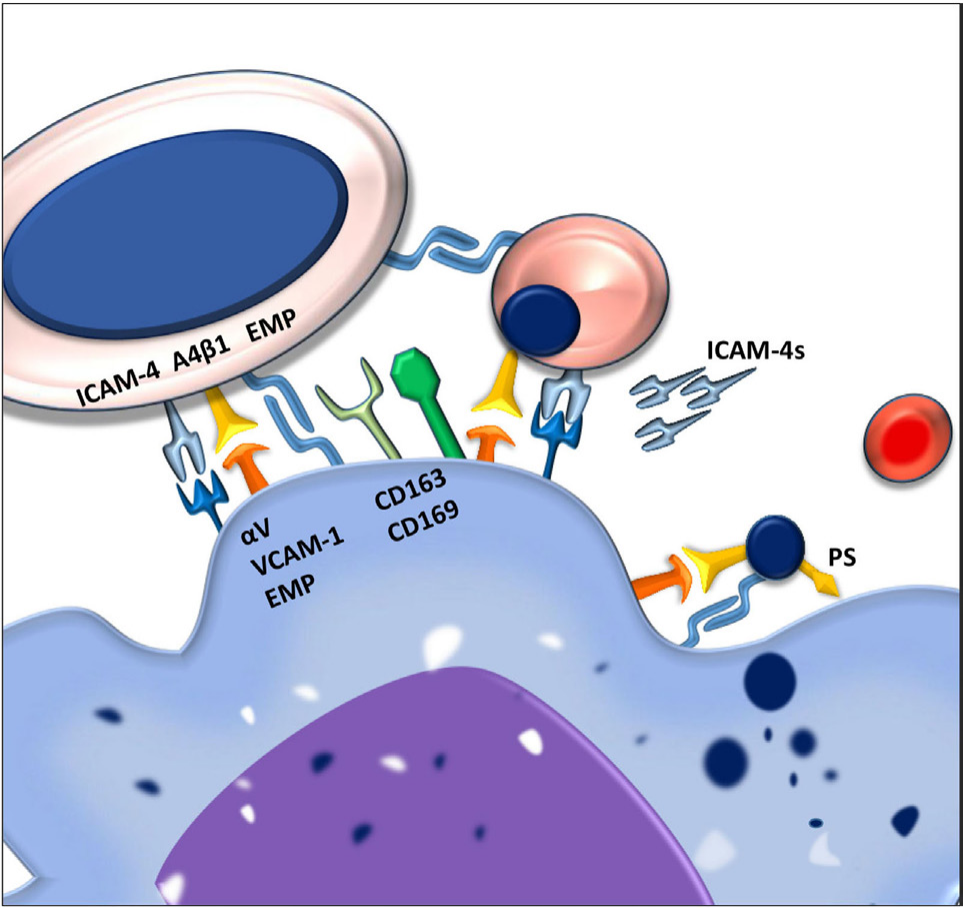
**Macrophages and erythroblasts form erythroblastic islands through adhesion molecules**. Macrophages express several adhesion molecules that facilitate interactions with erythroid precursors of various stages. The intercellular adhesion molecule 4 (ICAM-4) and the α_4_β_1_ integrin on erythroblasts interact with the α_V_β_1_ integrin and vascular cell adhesion molecule-1 (VCAM-1) on the macrophage. Gradual loss of the α_4_β_1_ integrin and EMP on the erythroblast facilitates the release of reticulocytes from the erythroblastic island. Persistence of these adhesion molecules on the pyrenocytes is contributing to its phagocytosis by the central macrophage.

Erythroblastic islands can be found in all tissue compartments that support erythropoiesis including the yolk sac, fetal liver, bone marrow, and in certain cases the splenic red pulp (5). They are important for proper erythropoiesis as depletion of bone marrow macrophages, or interference with the adhesion molecules that contribute to the formation of erythroblastic islands, leads to pro-erythroblast differentiation arrest, a reduction in proliferation, increased apoptosis, and ultimately anemia (6–8). Several adhesion molecules have been identified that are important for formation and stabilization of erythroblastic islands and thus for erythropoiesis (Figure 1).

One of the first adhesion molecules described to be important for erythroblastic island formation is erythroblast macrophage protein (Emp). Emp is expressed both on the central macrophage as well as on the erythroblast (9). The homotypic interaction between Emp on the macrophage and Emp on the erythroblast is one of the prerequisites for efficient erythropoiesis since interfering with this interaction, using blocking antibodies, decreases proliferation and results in increased erythroblast apoptosis (10). Even though macrophages have been shown to be able to interact with Emp-null erythroblasts, these erythroblasts do not enucleate, underlining the importance of this adhesion molecule in erythropoiesis.

Next to Emp, several other adhesion molecules are important in the formation of stable erythroblastic islands. These include the α_4_β_1_ integrin and intercellular adhesion molecule 4 (ICAM-4) expressed on erythroid precursors. These molecules interact with vascular cell adhesion molecule-1 (VCAM-1) and α_V_ integrins, respectively, such as α_V_β_1_ and α_V_β_5_ (11). By interfering with the interactions between these receptor pairs by use of blocking antibodies, it was found that these adhesion molecules are important for erythroblastic island integrity. Interfering with the ICAM-4–α_V_ integrin interaction was shown to lead to a reduction in erythroblastic island number as well as the total number of erythroblasts per island (6). Similarly, loss of α_4_β_1_-VCAM-1 interaction led to erythroblastic island disruption (8). Since abrogation of the ICAM-4–α_V_ integrin binding was found to lead to reduced interaction between erythroblasts and macrophages, and since ICAM-4 expression is decreasing during erythropoiesis, the discovery of a secreted form of ICAM-4, ICAM-4S, prompted the hypothesis that ICAM-4S is involved in the release of terminally differentiated erythroblasts from the erythroblastic island (12).

Apart from promoting erythroblastic island stability and formation, several adhesion molecules found on the central macrophages, such as CD163 and CD169, have been shown to promote proliferation. CD163 consists of nine so-called scavenger receptor cysteine rich (SRCR) domains. The third SRCR domain within CD163 functions to scavenge hemoglobin–haptoglobin complexes from the circulation (13). A 13 amino acid motif within the second scavenger domain of CD163 was found to directly interact with erythroblasts (14). It was furthermore established that CD163 interaction with erythroblasts promoted expansion but not differentiation. This was shown to be dependent on the maturation stage of the erythroblast as the interaction between macrophage CD163 and the erythroblast is quickly lost past the pro-erythroblast stage, indicating that its function is to regulate early pro-survival signals.

The counter receptor for CD163 on the erythroblast has not yet been identified. What is known is that upon interaction of CD163 with the hemoglobin–haptoglobin complex, a cascade of intracellular signaling is induced within the macrophage, including calcium mobilization and inositol triphosphate production. Subsequently, through an autocrine feedback loop, the secretion of interleukin-6 and macrophage colony-stimulating factor (CSF-1) leads to macrophage survival (15). It is of interest to assess whether these, or similar downstream signaling events, also occur after binding of erythroblasts and thus stimulate macrophage survival through CSF-1, indirectly aiding erythroblast expansion. Another macrophage adhesion molecule that is important for erythropoiesis is CD169 (16), a surface molecule originally identified as the “sheep erythrocyte receptor” or sialoadhesin, based on its capacity to recognize sialic acid residues (17). An interesting study by the group of Jacobsen showed the importance of CD169+ macrophages in erythropoiesis. In this study, treatment of mice with granulocyte colony-stimulating factor (G-CSF) was found to impair bone marrow erythropoiesis. Searching for the underlying cause they established that bone marrow macrophage numbers were greatly reduced. Characterization of bone marrow macrophages showed that CD169+ but not CD169− macrophages were lost upon G-CSF treatment (7). Currently, it is unclear why CD169+ macrophages in the bone marrow are mainly affected, especially since G-CSF treatment did not ablate CD169+ macrophages in the spleen. It was furthermore established that splenic CD169+ macrophages, upon CD169+ macrophage depletion from the bone marrow, could facilitate extramedullary hematopoiesis (EMH) in the spleen which is speculated to compensate for the G-CSF induced dyserythropoiesis in the bone marrow. In polycythemia vera (PV), where the Janus Kinase V617F mutation JAK2(V617F) is hyper sensitizing erythroid precursors to EPO, the depletion of CD169+ macrophages was found to reduce splenomegaly and reticulocytosis resulting in normalization of the erythroid compartment in mice (16). This finding indicates that erythropoiesis in PV is still sensitive to the stimulating activity of macrophages and identifies these macrophages as a potential therapeutic target to counteract the exaggerated erythropoiesis induced by the JAK2(V617F) mutation. Another chronic stress condition resulting in anemia is beta-thalassemia (18). In this disease, the absence or reduced expression of β-globin chains causes excess α-globin chains to precipitate in RBCs and precursors, leading to premature RBC destruction and ultimately anemia. High Epo levels here result in increasing erythroblast numbers. To facilitate the high rate of erythropoiesis, iron absorption is increased which in chronic situations leads to toxic iron overload. Ablation of CD169+ macrophages in a PV mouse model using clodronate liposomes broke this cycle of continuous-accelerated inefficient erythropoiesis. Already 40 h after clodronate treatment, an improvement of the anemia was observed (19). Within the bone marrow and spleen, the depletion of macrophages led to a reduction of erythroid progenitors and a proportional increase in differentiated erythroid cells. This clearly shows that although Epo/EpoR/JAK2 are master regulators in erythrocytosis in PV, CD169+ macrophages play an important role in the exaggerated levels of erythropoiesis during chronic stress conditions, which is ameliorated upon macrophage depletion. How exactly CD169+ macrophages exert this proliferative potential upon erythropoiesis is still not clear. What is known though is that CD169+ macrophages are pivotal in retaining HSCs in the bone marrow mesenchymal stem cell niche (20). Furthermore, also during later stages of hematopoietic development, CD169+ macrophages are important for erythroid proliferation. This was addressed in a study where through electron microscopy it was found that CD169+ macrophages induced loose interactions with erythroblasts leading to the formation of erythroblastic islands, allowing rapid proliferation and inducing pro-erythroblast cytokinesis (21). In contrast, CD169− macrophages engaged tight interactions with erythroblasts leading to erythroblast phagocytosis and impaired proliferation (21). How, mechanistically, CD169+ macrophages induce cytokinesis is still subject to investigation. However, an interesting candidate to direct future research toward is Mucin-1 as it was found to act as a ligand for CD169 (22). This heavily *O*-glycosylated protein is gradually expressed during erythropoiesis and found to be absent in RBCs. Similar to other known SIGLECs, the cytoplasmic tail of CD169 contains tyrosine motifs that induce intracellular signaling upon ligation. Upon ligation of CD169 by antibody crosslinking, clathrin-mediated endocytosis was observed (23). Current focus regarding CD169-mediated endocytosis involves toxins and lipid antigen uptake, as a means of antigen internalization and ultimately presentation. It would be of interest to determine how CD169 on macrophages contributes to proliferation of erythroblasts.

## Secretion of Soluble Factors Important in Erythroblastic Islands

Various soluble factors have been identified that are important in defined stages of erythropoiesis. Within erythroblastic islands, both macrophages and erythroblasts secrete, or are dependent on, soluble factors. Some of these factors have been found to be important for erythroblastic island integrity, but some also for proliferation, differentiation, and hemoglobinization of RBC precursors.

Macrophages and erythroblasts cross-stimulate each other to promote proliferation. Macrophages have, for example, been shown to secrete insulin-like growth factor-1 and bone morphogenetic protein 4 (BMP4) to stimulate BFU-e and CFU-e proliferation (24, 25). On the other hand, erythroblasts secrete vascular endothelial growth factor A placental growth factor and growth-arrest specific 6 that are thought to aid macrophage proliferation (26–29). Another factor that is important for erythropoiesis is ferritin. In splenic and bone marrow macrophages but also hepatocytes and Kupffer cells, excess iron is stored in this globular protein (30). It was found that bone marrow and spleen derived macrophages, in absence of transferrin, could synthesize ferritin and excrete it. Subsequently, erythroid precursors were found to internalize ferritin through endocytosis, surprisingly, through transferrin receptor-1 (31–33). Some findings suggest that macrophages directly supply ferritin to surrounding developing erythroblasts. Ferritin molecules were, for example, found to be localized between the membranes of the central erythroblastic island macrophage and the erythroblast (34). However, in mice, serum ferritin was found to contain low levels of iron. It is thus questioned whether serum-derived ferritin contributes, in any significant way, to hemoglobin synthesis in erythroid precursors (32).

Aside from factors positively influencing erythroid and macrophage proliferation, several secreted factors negatively regulate erythropoiesis. These include the cytokines transforming growth factor-β (TGF-β), tumor necrosis factor-α (TNF-α), and interferon-γ (INF-γ) (35–38). INF-γ and TNF-α negatively regulate erythropoiesis mainly through induction of apoptosis (37, 39). Production of TNF-α by macrophages, for example, induces caspase-mediated cleavage of transcription factor GATA-1 (37). GATA-1 is critical for pro-erythroblast differentiation. This is not only illustrated by the observation that pro-erythroblasts from chimeric GATA-1 knockout mice fail to mature beyond this stage and go into apoptosis (40) but also by individuals carrying mutations in the *GATA1* gene who suffer from dyserythropoietic anemia (41–43). Production of TGF-β by macrophages negatively regulates erythropoiesis quite differently. It was found that TGF-β markedly accelerated and increased erythroid differentiation but at the same time induces loss of proliferative potential in RBC precursors thereby causing erythropoietic arrest (36).

Besides positive and negative regulators of normal erythropoiesis, several secreted factors have been identified that are important during stress erythropoiesis, as is found during anemia due to trauma or sepsis. In stress erythropoiesis, RBC output by CFU-e cells is insufficient due to the limited amount of divisions these cells undergo. BFU-e progenitor cells in such conditions are therefore induced to form new CFU-e cells. Several factors have been identified that play a role during stress erythropoiesis. These factors include glucocorticoids (GC), BMP4, and stem cell factor (44–46). Mice lacking the GC receptor, for example, display normal erythropoiesis but are incapable of inducing stress erythropoiesis as they fail to induce CFU-e proliferation in the spleen as seen in wild-type mice (44). Similarly, BMP4 expression in the spleen, as a response to acute anemia, was found to drive differentiation of immature progenitors into so-called stress BFU-e cells, allowing rapid expansion and erythropoiesis (47).

## Macrophages Support Terminal Differentiation in Erythropoiesis

The last stages of erythroid development include the expulsion of the nucleus and membrane remodeling of the nascent reticulocyte in the circulation (48–50). Macrophages phagocytose expelled nuclei in the bone marrow and facilitate the maturation process of circulating reticulocytes in the spleen.

One of the key elements in the clearance of the expelled nuclei, the so-called pyrenocytes, by macrophages, is the sorting process of membrane proteins. During nuclear extrusion, proteins are selectively being directed toward either the reticulocyte or pyrenocyte membrane. Cytoskeletal proteins and integral membrane proteins important for RBC shape and deformability such as ankyrin, spectrin, and the glycophorins are sorted toward the reticulocyte membrane. On the other hand, proteins associated with cellular adhesion such as Emp1 and β_1_ integrins are sorted toward the pyrenocyte (48). Although it is currently unclear how this sorting process works, it is hypothesized that cytoskeletal interactions play an important role since protein sorting is defective in disorders such as elliptocytosis, spherocytosis, and sickle cell disease (51, 52). It is clear, however, that during terminal maturation, proteins in the RBC membrane undergo dramatic reorganization. The sorting of adhesive molecules Emp and β1 integrins toward the pyrenocyte allows for the interaction with macrophage Emp and VCAM-1, respectively. In addition, it is thought that a combination of ATP depletion and concomitant phosphatidylserine (PS) exposure signals the macrophage through one of its PS receptors to phagocytose the pyrenocyte (53).

Finally, reticulocytes are released into the circulation where they mature into RBCs. During this stage, reticulocytes lose volume, become biconcave, and acquire the typical flexibility of an RBC (54, 55). Furthermore, expression of the transferrin receptor is quickly downregulated through exosome release to prevent excessive iron import and toxicity (56). It is thought that red pulp macrophages (RPMs) of the spleen play an important role in this maturation process, as depletion of macrophages by clodronate treatment inhibits the maturation of reticulocytes in the circulation (57). How splenic macrophages come into contact with early reticulocytes and promote their maturation into RBCs is still not clear. One hypothesis involves a mechanism similar to recognition of aged RBCs, a process that is described more extensively below, and is based on the capacity of the red pulp of the spleen to filter out rigid RBCs. A characteristic that old RBCs and reticulocytes share is a relative lack of deformability. Treatment of healthy RBCs with agents that alter their deformability, such as chloroquine, leads to RBC accumulation in the spleen (58). Thus, reticulocytes may be trapped in the spleen by their lack of deformability, thereby coming into close proximity to the RPMs. A protein that seems to be involved in reticulocyte maturation and differentially regulates membrane stiffness of reticulocytes and RBCs is glycophorin-A (GpA). It was found that in reticulocytes GpA is strongly interacting with the underlying cytoskeleton and thereby largely contributes to membrane rigidity. In terminally differentiated RBCs, however, the interaction with the cytoskeleton is less prominent, resulting in a more deformable membrane (59). It is therefore postulated that the spleen acts as sieve where non-deformable cells are captured and that, here, macrophages await to facilitate reticulocyte remodeling.

## Role of Macrophages in EMH

Hematopoiesis occurring outside the bone marrow is referred to as EMH. In healthy mice, roughly 10% of erythropoietic output is facilitated by the spleen (60). In human spleen, although some hematopoietic elements are usually present, it is generally accepted that, during steady state, its contribution to hematopoiesis is negligible (61, 62). There are certain circumstances during which EMH occurs. During fetal development, for example, EMH occurs in the yolk sac, fetal liver, and spleen (63–65). In adulthood, bone marrow abnormalities including overproduction of marrow elements, damage to the bone marrow, or alterations in cellular bone marrow constituents can cause EMH. Also, prolonged hypoxic conditions, chronic inflammation and infection, and genetic disorders can contribute to EMH (66).

A commonly used agent to induce and study EMH is G-CSF. G-CSF treatment in mice leads to the depletion of macrophages from the bone marrow, by a mechanism that is still obscure. In these mice, erythropoiesis is arrested at the pro-erythroblast stage. It is well recognized that this causes EMH (67). It is believed that mobilization of HSC to the spleen, especially, when G-CSF treatment is combined with cyclophosphamide treatment is contributing to EMH (68). Macrophages play a key role in capturing the mobilized HSCs through vascular VCAM-1 (69).

Additional to retaining HSCs in the spleen, macrophages were found to play a central role in ongoing EMH. Hepatic sinusoidal macrophages were found to form erythroblastic islands upon induction of anemia in mice by injection of phenyl hydrazine (70). To assess the importance of CD169+ macrophages in EMH of the spleen and bone marrow, a mouse model was used where the diphtheria toxin receptor (DTR) was knocked-in downstream of the Siglec 1 promotor, a protein found specifically in macrophages. Treatment of these CD169DTR/+ mice with diphtheria toxin leads to ablation of these macrophages. Short exposure to DT resulted in CD169+ macrophage ablation from bone marrow but not from spleen. Using phenylhydrazine it was found that splenic VCAM-1, an adhesion molecule facilitating erythroblastic island interactions, on CD169+ bone marrow macrophages, was critical to recover from hemolytic anemia (16).

EMH can also be observed during infection. Systemic infection by *E. coli* in mice was found to lead to a 744-fold increase in G-CSF serum levels, directly inducing HSC mobilization to the spleen (71). Similarly, *S. aureus* was shown to induce production of IL-3 and GM-CSF in spleen cell cultures of mice (72). In hemaglobinopathies such as β-thalassemia and sickle cell disease, the premature destruction of RBCs and erythroid precursors causes anemia, overproduction of Epo and ultimately EMH in spleen and liver (73). Since in these diseases the erythroid progenitors are susceptible to apoptosis, increased Epo levels here do not compensate the anemia.

Taken together, disease, infection, and genetic disorders all can lead to EMH of which the underlying mechanism is not always clear. During anemia, the body attempts to increase erythropoiesis by excretion of hematopoietic factors and mobilization of HSCs to other tissues such as the spleen. Here, macrophages can trap HSCs through, amongst others, VCAM-1 and in the presence of the right factors initiate erythroblastic island formation and EMH. The relationship between anemia and EMH is intimate but still not well understood at the molecular level. Future studies may shed further light on the factors that drive EMH.

## Repair and Clearance of RBC by Macrophages in the Spleen

After their development in the bone marrow, RBCs have a life span of approximately 120 days (74), during which they have numerous interactions with macrophages of the spleen and liver. With time the plasma membrane of the RBC undergoes deleterious changes that make the cell susceptible to clearance by macrophages (75). The spleen is the primary organ to filter effete RBC out of the blood. It is anatomically divided in red pulp, which is responsible for the filtering function, and white pulp, which is dedicated to adaptive immunity. The red and white pulp are divided by the marginal zone (76). With its specialized structure of the venous system, the red pulp of the spleen has the unique capacity to retain old RBC that has become less deformable (74, 75). The membrane of RBCs is extremely elastic. This deformability is of great importance since it allows RBCs to pass through capillaries that are narrower than their own diameter (77, 78). Over time, RBCs lose their elasticity. RPMs located in the cords of the red pulp phagocytize the RBCs that are too rigid to pass through the inter-endothelial slits of the red pulp (74). The high percentage of macrophages in the red pulp and the unique structure of the spleen give it its specialized ability to scrutinize the integrity of RBCs (75). Decreased RBC deformability is not only a characteristic of senescent RBCs (79). In many pathological conditions such as sickle cell disease, hereditary spherocytosis, or sepsis RBC clearance is enhanced due to rigidification of the RBC membrane (80–84). Stiffening of the RBC membrane is also observed in malaria which is discussed below. On the opposite side, hyperactivation of macrophages may also lead to enhanced clearance. This can occur for instance in the hyperinflammatory syndromes, hemophagocytic lymphohistiocytosis (HLH) (85). HLH is a rare disorder that is characterized by spiking fevers and hemophagocytosis by activated macrophages (86). It can be caused by various disorders which can be hereditary or secondary to several pathologies such as infection, malignancy, or autoimmune disease (87). Depending on the background of the disease, numerous mechanisms have been proposed to explain hemophagocytosis in HLH. It is suggested that in most if not all forms of HLH, the hyperactivation of macrophages is caused by a cytokine storm induced by uncontrolled activated NK cells and cytotoxic T-lymphocytes (88).

In addition to inspecting RBC membrane deformability, macrophages also remove inclusion bodies from circulating RBCs (89). Already in the late 1950s, it was shown that this process is exclusively executed in the spleen. Crosby et al. showed that injected-labeled siderocytes (RBCs containing iron filled granules) in subjects with a spleen decreased over time while in splenectomized subjects the amount of siderocytes remained unchanged (89). Moreover, splenectomized patients show a high amount of RBCs with Howell-Jolly bodies (nuclear chromatin inclusions), Heinz bodies (inclusions of denatured hemoglobin), siderocytes, and Pappenheimer bodies (inclusions formed by phagosomes that have taken up excessive amounts of iron) (90). Hence, it seems that the spleen and its residing RPMs not only clear senescent RBCs but are also responsible for keeping RBC “healthy.” RBCs contain a variety of enzymes to protect the cell from oxidative damage. Yet, RBCs are not capable of synthesizing new proteins and in time oxidative damage will accumulate resulting in the formation of inclusion bodies (91). The exact mechanism underlying the removal of these inclusion bodies is not known, but it has been proposed that RBC vesiculation is involved in the disposal of RBC damage (92).

## Red Pulp Macrophages

The RPMs are extremely potent in neutralizing the toxic effects of hemoglobin. They express high levels of the hemoglobin scavenger receptor CD163 and the enzyme heme-oxygenase 1 (HO-1), which plays a crucial role in heme degradation (13, 76, 93). Recycling of iron through erythrophagocytosis by macrophages is the largest contributor to iron homeostasis. After phagocytosis of senescent RBCs, hemoglobin is degraded into heme and iron after which it is exported through ferroportin into the plasma. Here, transferrin traffics iron to cells that require iron including developing erythroid cells in particular [reviewed in Ref. (94)]. HO-1 is one of the critical enzymes in this cascade. Its absence leads to splenic and liver macrophage apoptosis and concomitant release of non-metabolized heme. In the spleen, this leads to red pulp fibrosis, atrophy, and ultimately functional aspleny (95).

The origin of RPMs is a current point of discussion. Recent studies by Geissmann et al. suggest that primitive macrophages originate from the yolk sac (96). However, subsequent studies propose that fetal HSCs give rise to RPMs (76, 97–99). The general view is that in the homeostatic situation macrophage populations are maintained by local proliferation. Nonetheless, in pathological conditions, such as infection and inflammation, circulating monocytes may enter the spleen and differentiate into RPMs (99–101). It seems that in inflammatory situations, the “steady-state” macrophage populations are substituted for an inflammatory monocyte derived pool of macrophages. Besides macrophages also other phagocytes may contribute to RBC clearance in pathological situations. Stijlemans et al. show that in mice trypanosomiasis, a parasitic disease, increases erythrophagocytosis by neutrophils and monocytes (102). This indicates that besides macrophages other phagocytes can play a role in RBC clearance during infection.

## Clearance of Blood-Borne Infections in the Spleen

The primary function of RPMs is believed to be the scavenging of senescent RBCs. Nevertheless, besides their homeostatic role, RPMs are important to control blood-borne diseases through their ability to control iron availability for infecting pathogens. Several diseases are associated with loss of RPMs due to for instance auto-splenectomy as observed in SCD. A combination of factors causes functional asplenism in these patients. First, slow RBC circulation speeds and high oxygen extraction rates cause the RBCs to sickle. Mechanical obstruction by sickled RBCs but also adhesion of RBCs to endothelium obstructs red pulp sinuses of the spleen and can contribute to infarction and in the long-term functional aspleny (103, 104). Since macrophages in the spleen play a key role in removing both senescent RBCs as well as blood-borne pathogens from the circulation, the functional aspleny causes these patients to become more susceptible to blood-borne infections. Moreover, in SCD and thalassemia patients, or in patients suffering from iron overload in general, infection by pathogens such as *Y. enterocolitica* but also to streptococci is often observed (105, 106). Besides taking up whole RBC, macrophages can also take up immune complexes or pathogens bound to complement receptor 1 on RBCs while leaving the RBC intact, a process termed immune adherence clearance (107).

Red pulp macrophages are suggested to be specifically important in the control of *Plasmodium*-infected RBCs. Malaria is the most frequently acquired infection affecting RBC and has a major impact on RBC deformability (75). *Plasmodium* parasites have a complex life cycle that starts off in hepatocytes and is followed by asexual stages in the RBC. This involves major restructuring of the RBC membrane and cytoskeleton (108). While the young asexual stages do not impact RBC deformability to a great extent, the mature asexual stages of the parasite cause stiffening of the RBC membrane, which makes them susceptible for removal in the spleen (75). Splenectomized mice show increased susceptibility toward *Plasmodium chabaudi* infections (109). Moreover, Couper et al. show that macrophage depletion in *Plasmodium yoelii*-infected mice results in exacerbated parasite growth and more profound anemia (110). These findings indicate a vital role of macrophages in the spleen in malaria infections. Although it is generally believed that RPMs play a central role in control of malaria infections, the extent of their importance is debatable. Studies show that parasitized RBCs are found in the red pulp (109), RPMs can remove parasites internalized by the RBC while leaving the RBC intact, a process called pitting (111), and RPMs expand during malaria infections (112). However, Kim et al. show that while RPMs produce large amounts of type I interferons during *P. chabaudi* infections *Spic^−/−^* mice, which lack RPMs completely, eliminate the parasite as efficiently as Wt mice (112). It was concluded that RPMs contribute to early immune infection recognition and activation, while they may be dispensable for control of the infection due to compensation by infiltrating monocytes.

## RBC Clearance in the Liver

Besides the spleen also the liver is an important RBC depot and has an important role in RBC clearance and iron recycling (102, 113, 114). Current research suggests that under pathological conditions the liver and not the spleen is the primary site of RBC clearance. Theurl et al. show in a mouse model that infused-stressed RBCs predominantly end up in the liver (114). The presence of stressed or damaged RBCs led to a rapid recruitment of monocytes. These monocytes give rise to “transient macrophages” that are well equipped to phagocytose RBCs and recycle the iron. This specific macrophage population was only found in the liver, and the researchers suggest that therefore the liver is uniquely adapted to respond to an increased demand for RBC phagocytosis. This hypothesis is supported by the work of Stijlmans et al. in which it is shown in mice that LPS injection or *T. brucei* infection leads to enhanced erythrophagocytosis primarily in the liver (102).

## Band 3-Mediated Clearance of RBC by Macrophages

**Figure 2:**
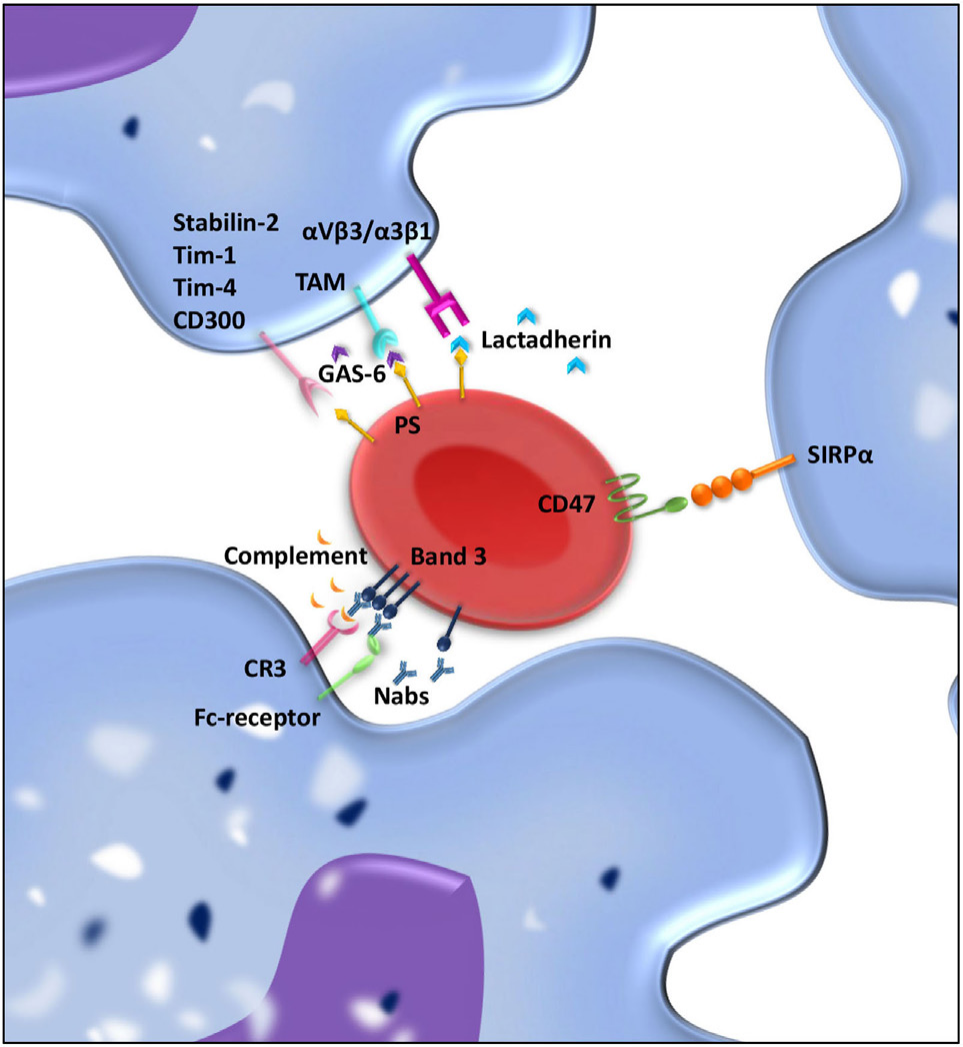
**“Eat me” and “don’t eat me” signals involved in interaction between macrophages and red blood cell (RBC) regulating clearance**. RBCs and macrophages interact with each other through ligand–ligand interactions. Over time, “eat me” signals accumulate on the RBC membrane. Phosphatidylserine (PS) exposed on the RBC membrane can directly bind Stabilin-2, Tim-1, Tim-4, or CD300 on the macrophages and is presumed to give a pro-phagocytic signal. Moreover, bridging molecules such as GAS-6 and lactadherin can facilitate RBC-macrophage interaction by binding PS on RBCs and TAM receptors or α_v_β_3_/β_5_ integrins on the macrophage. Band 3 clustering and opsonization with Nabs and complement on the RBC enables binding to the macrophage *via* Fc receptors and CR-1 and thereby facilitates phagocytosis. As a counterbalance, CD47–SIRPα interactions inhibit phagocytosis of RBCs by the macrophage.

Red blood cell clearance can be viewed as a two component process. On the one hand, mechanical retention and RBC deformability determine if an RBC gets cleared from the circulation; on the other hand, cell biological factors are involved in RBC removal through erythrophagocytosis in the spleen. So-called “eat me” signals accumulate on the cell membrane of the RBC over time, and these signals can trigger RBC clearance by macrophages (Figure 2) (115–117). There is no clear consensus about what these “eat me” signals exactly are (113). Yet, there have been several proposed mechanisms of RBC clearance. Firstly, it has been suggested that band 3, a highly abundant transmembrane protein, is the main target of natural occurring antibodies (Nabs) (116, 118, 119). Nabs are antibodies present in the serum of healthy individuals that have been formed without the apparent antigen exposure, for instance, anti-A antibodies in B-positive individuals (120). These immunoglobins are preferentially of the IgM isotype, and their production already starts before birth by B cells of the B1 type in the marginal zones of the spleen (121). Nabs recognize self-, altered-self, and foreign antigens. Besides protecting from invading pathogens they are important regulators for clearance of necrotic or apoptotic cells (122). Over time, band 3 is believed to be modified mostly due to oxidative damage, which leads to the formation of epitopes for Nabs binding (118, 123). The exact nature of epitope formation allowing Nab binding is still under debate. It is hypothesized that oxidative damage to hemoglobin leads to the formation of hemichromes (hemoglobin denaturation products), which bind to band 3 and cause band 3 clustering. This in turn results in Nab binding (124). An alternative hypothesis for band 3 epitope recognition by Nabs is proteolytic degradation of band 3 (119). Kay et al. showed that IgG eluted from scenescent RBCs rebound to proteolytic fragments of band 3. This led to the hypothesis that a cell-age-specific antigen is exposed by a proteolytic modification (125). Due to their low affinity and low numbers in the circulation, Nabs are not efficient opsonins. It has been proposed that erythrophagocytosis can be enhanced by complement activation through the classical pathway after opsonization of RBCs with Nabs (116). Complex formation between Nabs and complement C3 fragments is potent opsonins that are readily recognized by complement receptors on macrophages (and other phagocytes) (123, 126, 127).

## Oxidative Stress/Band 3-Mediated Clearance in Disease

In the homeostatic situation, RBC removal and erythropoiesis is balanced. In pathological situations, the oxidative insults modifying band 3 can be rapid and massive leading to accelerated RBC removal. Glucose-6-phosphate dehydrogenase (G6PD) deficiency is an example of a genetic RBC defect that exhibits insufficient antioxidant defense and hence anemia due to enhanced RBC destruction (118). G6PD is an enzyme involved in the production of NADPH, a cofactor used in anabolic reactions. NADPH is crucial for the protection of the cell against oxidative stress (128). Due to its role as oxygen carrier, RBCs are particularly exposed to oxidative damage. G6PD patients therefore have an increased risk for RBC destruction due to oxidative stress which can be triggered by medication, illness, or certain foods such as fava beans. In addition to RBC defects affecting antioxidant defense hereditary, RBC disorders such as thalassemias and hemoglobinopathies also show increased oxidative stress (129, 130). Besides inadequate ROS detoxification free heme and iron may as well give rise to enhanced oxidative stress since partially oxygenated hemoglobin is susceptible to redox reactions (118, 131). In all these diseases, high levels of anti-band 3 antibodies and C3b are found bound to RBCs (132, 133). Increased RBC phagocytosis is observed which can be ameliorated in many patients by a splenectomy (124).

Besides their role in genetic RBC defects, anti-band 3 antibodies are also said to be important in RBC clearance during malarial infections (115, 134, 135). Infected RBCs demonstrate hemichrome binding to band 3 followed by band 3 clustering and opsonization with Nabs and complement (135, 136). Sickle cell trait, thalassemia trait, and G6PD-deficient individuals are known to have a selective advantage with respect to malarial infections. RBCs of these patients are more sensitive to oxidative stress exerted by the malaria parasite (124). Already in early stages of the infection, RBCs are opsonized with Nabs, which may be one of the many reasons that malaria infections are more efficiently cleared in these patients.

## PS-Mediated RBC Clearance

A second mechanism that has been proposed to be important for RBC removal is PS exposure at the cell surface of the RBC. In healthy RBCs, PS is located on the inner leaflet of the RBC membrane (137). On the outer leaflet of the RBC, PS exposure is suggested to be an “eat me” signal that triggers phagocytosis by macrophages. PS exposure can be induced by an increase in cytosolic Ca^2+^ concentration, which can be a result of oxidative stress, osmotic shock, or other forms of cell stress (138). PS exposure as a signal for clearance is not restricted to RBCs but is seen as a general removal signal of senescent or damaged cells. In nucleated cells, PS exposure is part of the apoptotic process. Since RBCs are non-nucleated cells, apoptosis does not occur, but the regulated process of cell death in RBC has been given the term eryptosis (139). PS-dependent apoptotic cell phagocytosis is evolutionary conserved and not only found in mammals but also in lower organisms such as *C. elegans* and *Drosophila* (140). Macrophages display an array of receptors for PS including Tim-1, Tim-4, Stabilin-2, and CD300 (141–144). Furthermore, there are various bridging molecules, which can bind to PS on the cell surface, such as lactadherin, GAS-6, and protein S. These proteins than facilitate binding to α_v_β_3/5_ integrins or TAM receptors, respectively, on the macrophage and thereby induce clearance of PS-exposing RBCs (145).

## PS-Mediated RBC Clearance in Disease

There is a multitude of diseases associated with PS-mediated RBC clearance. Some examples of pathologies where increased oxidative stress activates Ca^2+^ cation channels and thereby induces PS exposure and eryptosis are sickle cell anemia, thalassemia, G6PD, iron deficiency, chronic kidney disease, and diabetes (138). Other diseases that are postulated to result in eryptosis are malignancies, cytostatic drug-induced anemia, hepatic failure, heart failure, and dehydration (138). PS exposure induced by the malaria parasite has been widely investigated. To ensure pathogen survival, *Plasmodium falciparum* activates oxidant-sensitive ion channels in the RBC membrane by inducing oxidative stress. These channels facilitate cellular uptake of nutrients, Na^+^ and Ca^2+^, and waste removal, which are crucial for parasite survival, yet a secondary concequence is PS exposure (146, 147). It has been suggested that *P. falciparum* prolongs RBC lifespan by sequestrating Ca^2+^ and digesting hemoglobin to diminish RBC swelling and preserve osmotic stability in the RBC (138, 148). The genetic resistance toward malaria infections found in sickle cell trait, thalassemia trait, and G6PD-deficient individuals has partially been attributed to the sensitivity of their RBC to induce PS exposure and erypotosis upon infection with as a result accelerated clearance of infected RBCs (149).

## CD47-Mediated RBC Clearance

Although there is still debate on the exact nature of “eat me” signals on RBC membrane, it has been generally accepted that CD47 is a “don’t eat me” signal that plays a crucial role in RBC homeostasis (150, 151). CD47 is a ubiquitously expressed protein that binds the inhibitory receptor signal-regulatory protein alpha (SIRPα) present on macrophages but also other myeloid cells. The CD47–SIRPα interaction inhibits immune responses such as phagocytosis (152). The strong negative signal for phagocytosis brands CD47 a marker of “self” (150). Besides functioning as a “don’t eat me” signal, our group has shown that a conformational change in the CD47 protein can switch the molecule from a “don’t eat me” to an “eat me” signal (117). This conformational change in CD47 can be induced by oxidative stress and promotes TSP-1 binding to CD47, which creates a novel binding site for SIRPα. This alternative binding site for SIRPα induces a pro/phagocytic signal for the phagocyte. The dual role of CD47–SIRPα in regulating RBC uptake demonstrates the complexity of RBC clearance (117).

## CD47-Mediated RBC Clearance in Disease

The role of CD47 as a “don’t eat me” or as an “eat me” signal in RBC clearance in pathological conditions is still rather unclear. It has been suggested that anemia in untreated Gaucher disease can be partly explained by reduced expression of CD47 on RBCs (153, 154). In mice, it has been shown that CD47–SIRPα interactions have a profound effect on disease severity of autoimmune hemolytic anemia (AIHA) (155). However, in human, AIHA patients’ CD47 expression is normal (156, 157). In sickle cell disease, CD47 expression is not significantly altered in patients compared to healthy controls. Nonetheless, hydroxyurea, a commonly used therapy, induces overexpression of CD47 on RBCs (158). How CD47 expression is influenced in human infection models is mostly unknown. Yi et al. showed that the absence of CD47 on RBCs may be important for the process of detecting and mounting an immune response against infected RBCs. They showed that the lack of CD47 on RBCs activates dendritic cells which in turn stimulate CD4+ T cells and antibody responses (159). Chambers et al. show that *in vitro* exposure of RBC to *P. falciparum* has no effect on CD47 expression (160). Yet, interfering with the CD47–SIRPα interaction might be a promising therapeutic strategy in the control of malaria infections. Ayi et al. show that disrupting the CD47–SIRPα interaction enhances phagocytosis of infected RBCs (161). Moverover, Banerjee et al. demonstrated that *Plasmodium yoelii* preferentially infects RBCs expressing high levels of CD47 and that CD47^−/−^ is highly resistant to *P. yoelii* infections (162). In contrast to Chambers et al., these authors do find reduced CD47 expression on human *P. falciparum*-infected RBCs. All in all, the role of CD47 in pathological situation is still largely unknown. It is apparent that RBC clearance is a complex and multifactorial process that can be influenced by numerous factors such as, inflammation, infection, chemical substances, oxidative stress, or osmotic imbalance. Eventually, the balance between “eat me” and “don’t eat me” signals will determine if an RBC gets cleared from the circulation or not.

## Concluding Remarks

The interplay between macrophages and RBC shapes RBC formation, repair, and clearance. The intricate molecular mechanisms driving these processes are only partially understood. These mechanisms are plastic and differ greatly between steady-state and pathological conditions. In SCD, thalassemia, or hemolytic anemia, the body cannot always cope with the adverse effects observed. Modulating the interaction between macrophages and developing erythroblasts has already been shown to lead to favorable results. An example of this is the study by Chow et al. where depletion of CD169+ macrophages was found to normalize erythropoiesis in PV (16). Therefore, studying alternative mechanisms to fine-tune erythropoiesis and erythrophagocytosis in disease is important in reducing the clinical symptoms of several RBC-related diseases.

## Author Contributions

SM and TK wrote the review and designed the figures. TB and RB supervised and edited the manuscript.

## Conflict of Interest Statement

The authors declare that the research was conducted in the absence of any commercial or financial relationships that could be construed as a potential conflict of interest.

## Abbreviations

AIHA, autoimmune hemolytic anemia; BFU-e, burst-forming unit-erythroid cells; BMP4, bone morphogenetic protein 4; CFU-e, colony-forming unit-erythroid cells; CSF-1, macrophage colony-stimulating factor; DT, diphtheria toxin; EMH, extramedullary hematopoietis; Emp, erythroblast macrophage protein; Epo, erythropoietin; EpoR, erythropoietin receptor; G6PD, glucose-6-phosphate dehydrogenase; GAS-6, growth-arrest specific 6; GC, glucocorticoids; G-CSF, granulocyte colony-stimulating factor; GM-CSF, granulocyte-macrophage colony-stimulating factor; GpA, glycophorin-A; HO-1, heme-oxygenase 1; HSC, hematopoietic stem cell; ICAM-4, intercellular adhesion molecule 4; INF-γ, interferon-γ; IGF-1, insulin-like growth factor-1; IL-3, interleukin-3; Nabs, natural occurring antibodies; PGF, placental growth factor; PS, phosphatidylserine; PV, polycythemia vera; RBC, red blood cell; SIRPα, signal-regulatory protein alpha; SRCR, scavenger receptor cysteine rich; TGF-β, transforming growth factor-β; TNF-α, tumor necrosis factor-α; TRF-1, transferrin receptor-1; VCAM-1, vascular cell adhesion molecule-1; VEGF-A, vascular endothelial growth factor A.
